# Targeting Neuroblastoma Cell Surface Proteins: Recommendations for Homology Modeling of hNET, ALK, and TrkB

**DOI:** 10.3389/fnmol.2017.00007

**Published:** 2017-01-20

**Authors:** Yazan Haddad, Zbyněk Heger, Vojtech Adam

**Affiliations:** ^1^Department of Chemistry and Biochemistry, Mendel University in BrnoBrno, Czechia; ^2^Central European Institute of Technology, Brno University of TechnologyBrno, Czechia

**Keywords:** neuroblastoma, targeted therapy, homology modeling, norepinephrine transporter, anaplastic lymphoma kinase, neurotrophic tyrosine kinase receptor

## Abstract

Targeted therapy is a promising approach for treatment of neuroblastoma as evident from the large number of targeting agents employed in clinical practice today. In the absence of known crystal structures, researchers rely on homology modeling to construct template-based theoretical structures for drug design and testing. Here, we discuss three candidate cell surface proteins that are suitable for homology modeling: human norepinephrine transporter (hNET), anaplastic lymphoma kinase (ALK), and neurotrophic tyrosine kinase receptor 2 (NTRK2 or TrkB). When choosing templates, both sequence identity and structure quality are important for homology modeling and pose the first of many challenges in the modeling process. Homology modeling of hNET can be improved using template models of dopamine and serotonin transporters instead of the leucine transporter (LeuT). The extracellular domains of ALK and TrkB are yet to be exploited by homology modeling. There are several idiosyncrasies that require direct attention throughout the process of model construction, evaluation and refinement. Shifts/gaps in the alignment between the template and target, backbone outliers and side-chain rotamer outliers are among the main sources of physical errors in the structures. Low-conserved regions can be refined with loop modeling method. Residue hydrophobicity, accessibility to bound metals or glycosylation can aid in model refinement. We recommend resolving these idiosyncrasies as part of “good modeling practice” to obtain highest quality model. Decreasing physical errors in protein structures plays major role in the development of targeting agents and understanding of chemical interactions at the molecular level.

## Introduction

The large number of experimentally determined and deposited protein structures in the databases is a valuable source to explore uncharted territories of other proteins, which have similar sequence. Homology modeling, also known as comparative modeling, is used for constructing protein models based on template structures of homolog proteins. In this perspective article, we have highlighted the main structural issues in homology modeling of three candidate cell surface proteins for neuroblastoma targeted therapy. In the next section, we emphasize the challenges faced in each step in the homology modeling process. A brief section on targeting neuroblastoma describes the role of cell surface protein targets in developing therapeutics. A detailed evaluation of homology models expands on three chosen cell surface protein candidates: human norepinephrine transporter (hNET), anaplastic lymphoma kinase (ALK), and neurotrophic tyrosine kinase receptor type 2 (NTRK2, commonly known as TrkB). While the technical aspects of this manuscript favor an audience of specialists working on drug discovery and homology modeling of these three targets, the same principles of model evaluation and challenging issues can be applied for other homology models.

## Homology Modeling

Protein structures are the final frontiers in understanding the human biology at the molecular level. Gold standards of crystal structure characterization at atomic resolution, such as X-ray crystallography and nuclear magnetic resonance (NMR) spectroscopy, can be now satisfactorily complemented with computational methods (Kundrotas et al., 2012). Homology modeling is a systematic computational process where the most similar protein sequence, of a known crystal structure, is used for construction of a new model by replacing the equivalent amino acids on an equivalent backbone. Krieger et al. (2003) described seven steps in homology modeling: **(1) *The choice of template(s) and initial alignment*** are the first challenges in homology modeling. The accuracy of a homology model is correlated with the number of matching residues in alignment. A minimum 25% sequence identity has been the standard for homology modeling so far. Below 25% identity it is recommended to use multiple templates for modeling. The quality of template structure is directly inherited in the homology model. Polishing of template structure is a good practice before use. Missing atoms should be fixed using rotamer library and the hanging termini can be trimmed. **(2) *Alignment correction*** reduces errors caused by false sequence identities. Multiple alignments and structural alignments (e.g., position-specific scoring matrices) are recommended alternatives to conventional alignments. This step is the most critical in homology modeling before **(3) *backbone generation,*** as it determines the torsional angles of the backbone in the model. It has been shown that problems in the backbone can drastically alter the correct folding of the side-chains as well (Al-Lazikani et al., 2001). Issues regarding template recognition and alignment will be highlighted here. From our experience, many of the sporadic errors in modeling backbones arise in proline or adjacent to proline residues. **(4) *Loop modeling*** is important for correcting the folding of low-conserved regions (i.e., loops) of protein. It is now possible to make accurate models using database search or *ab initio* methods for up to 8–13 residues long loops (Totrov, 2012). Loop modeling employs potential energy scores for evaluation of the quality of constructed loop(s). It is also possible to use alternative rationales for evaluation based on biological functions. We show here some examples of biological functions that can include, exclude or guide the modeling process such as hydrophobicity, accessibility of loop residues to glycosylation or to binding of ions. **(5) *Side-chain modeling*** is directly affected by template sequence identity, alignment and backbone. Identical residues in two homolog proteins have nearly identical rotamers. The SWISS-MODEL server (Bordoli et al., 2009) and Modeller (Webb and Sali, 2014) are among the most commonly used platforms for backbone/side-chain homology modeling. Homology models are subjected to final optimization and validation before conducting various kinds of computational studies such as energetics (molecular mechanics), protein-drug/protein-protein interactions (molecular docking and protein networks), mutational analysis, and simulation in physiological environment (molecular dynamics). **(6) *Model optimization,*** also known as energy minimization, is application of energy functions to compute a global minimum that represents the most native folding (Bordner, 2012). Minimization aims to adjust the geometries of protein structures to the “force field” parameters used in computational studies. This method sometimes referred to as “relaxing the structure” is sufficient to resolve atomic clashes in the model. However, the story is not finished here. Several physical and structural errors should be resolved. **(7) *Validation*** of the final model(s) checks if the model complies with standard parameters of protein structure. These parameters include: bond lengths, bond angles, torsions, backbone outliers, rotamer outliers and all atomic contacts (Chen et al., 2010). Evaluation procedure includes physics-based, knowledge-based and experimental-based methods (Haddad et al., 2016). The same rigorous rules of evaluation that are applied in crystallography must also be applied in homology modeling.

## Targeting Neuroblastoma

Neuroblastomas are one of the most common and fatal solid tumors in children below 2 years of age. Whereas the survival rates of most types of cancer in children have improved in the past few decades, neuroblastoma is still below 75% 5-year survival (Siegel et al., 2016). Targeted therapy is a promising approach for developing treatments of neuroblastoma. Matthay et al. (2012) described three successful therapeutic targets for neuroblastoma that are in practice today: **(1)** hNET targeted by radiotherapy via ^131^I-metaiodobenzylguanidine (MIBG) **(2)** the GD2 ganglioside targeted by monoclonal antibodies, and **(3)** ALK targeted by kinase inhibitors. An extended list of targets was previously compiled by Verissimo et al. (2011), including those in clinical trials. The vast majority of FDA-approved medicines, particularly those discovered by rational drug design on molecular targets, work by perturbing the function of targets to prevent or reverse symptoms/disease (Kinch et al., 2015). It is important to distinguish the term *targeted therapy*, which occasionally indicates perturbing the molecular target, from the general concept of *targeting* which also includes drug delivery by recognizing cancer cells. Hence, *targeting* also describes therapeutics able to deliver and release cargo to the tumor microenvironment by binding to targets on the cancer cell surface (Shin et al., 2014). Such therapeutics (e.g., peptides and antibodies) are characterized by their selectivity to targets. They can be developed either blindly via screening methods or by molecular modeling. The ideal target should be “abundant and accessible and should be expressed homogeneously, consistently and exclusively on the surface of cancer cells” (Scott et al., 2012). Homology modeling has been successfully used in structure-based design of many therapeutics targeting different types of diseases including cancer (Butler et al., 2010; Schlessinger et al., 2011; DeVore and Scott, 2012). However, it is worth mentioning two categories of excellent protein targets in neuroblastoma that are beyond the scope of homology modeling: first, proteins which have known crystal structures such as CD147, which is associated with decreased neuroblastoma differentiation (Garcia et al., 2009; Wright et al., 2014), and CD57, which has also been implicated in aggression of neuroblastoma (Kakuda et al., 2004; Schlitter et al., 2012). Second, those candidate targets which lack any template structure. One of these cases is a glycoprotein called CD133, and its expression is associated with poor prognosis of neuroblastoma (Sartelet et al., 2012).

In comparative modeling for development of drugs, the lesson learned is that similar structures often exhibit similar functions. This is problematic particularly when drugs target the protein and its homologs simultaneously, resulting in various side effects. The three targets hNET, ALK and TrkB are good examples of cross interactions resulting in side effects. While hNET neurotransmitter transport overlaps with other monoamine transporters, the kinase inhibitors of ALK and TrkB can target their homologs, respectively. Both homology modeling and development of selective drugs should focus on the low-conserved regions of targeted proteins.

## hNET

**Figure 1:**
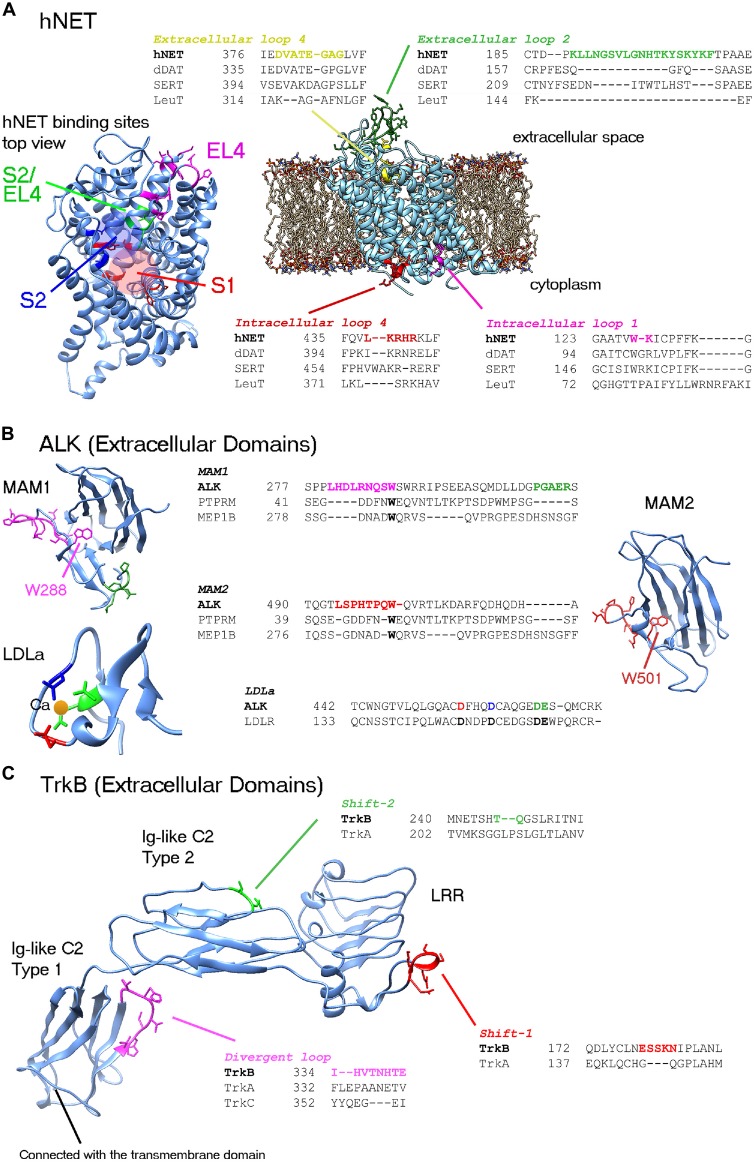
**Representations of the human norepinephrine transporter (hNET), anaplastic lymphoma kinase (ALK) and tropomyosin receptor kinase B (TrkB) structures featuring the most divergent regions which require precise alignment and possibly loop modeling.** Divergent loops are shown in representative alignments with closest known homolog structures. **(A)** hNET. Two divergent extracellular loops and two intracellular loops are shown. A top view of hNET is shown on the left. Low-conserved residues in binding sites as described by Andersen et al. (2015) are highlighted in colors: central binding site (S1), secondary binding site (S2) and extracellular loop 4 (EL4). Red and blue circles highlight S1 and S2 sites, respectively. **(B)** ALK extracellular domains. Conserved tryptophan in the MAM domains must be embedded in the center of structure (i.e., W288 and W501). Since the alignment of MAM2 (region of W501) was corrected manually, further loop modeling of the region is required (particularly for P496 and P499). Highlighted conserved residues that are binding the calcium ion in the Low-density lipoprotein receptor class A (LDLa) domain are important for alignment. **(C)** TrkB extracellular domains. Three major domains of the TrkB structure can be used in analysis. The leucine rich repeat (LRR) and Ig-like C2 Type 2 regions can be constructed by homology modeling. Two shifts are shown, that might require loop modeling. It is possible that divergent loops between Trk proteins might play role in the selectivity and cross-interactions with neurotrophins. The Ig-like C2 type 1 domain, directly connected to the transmembrane domain, has been illustrated by X-ray crystallography studies for the three Trks. Fitting of the three Trks showed a divergent loop at TrkB 334–341 region; possibly playing role in the selectivity of these Trks to different neurotrophins.

The hNET regulates the uptake and recycle of norepinephrine in the neurons. hNET is one of the highly expressed proteins in neuroblastoma (~90% of cases) and most commonly used in MIBG-based diagnosis/therapy (Brodeur et al., 1993; Matthay et al., 2012) and development of new therapeutics (Mortensen and Kortagere, 2015). hNET protein has three isoforms. The canonical isoform is 617 residues in length yet it is second to the longest isoform which is 628 in length and differs in the *C*-terminus. Previous homology models of hNET relied on the crystal structure of prokaryotic leucine transporter (LeuT; Yamashita et al., 2005) and *drosophila* dopamine transporter (dDAT) crystal structures as templates (Penmatsa et al., 2013; Wang et al., 2015; Table 1). Two recently published mutant serotonin transporter (SERT) crystal structures (Coleman et al., 2016) designated ts2 and ts3 can also aid in homology modeling of hNET. The homology models constructed by Schlessinger et al. (2011) and Koldsø et al. (2013) were based on LeuT template. In light of newly published dDAT and SERT crystal structures, we have recently addressed the developments regarding homology modeling of hNET based on these new templates (Haddad et al., 2016). Briefly, there are four major alignment gaps characterizing the sequences of dDAT, hNET and SERT; including the glycosylated extracellular loop 2 (EL2), extracellular loop 4 (EL4) and two intracellular loops (Figure 1A). Loop modeling is mostly required at the EL2 loop; particularly residues 189–207. After construction of a number of loops, one should exclude those with non-embedded leucines/valine residues due to their hydrophobicity. At first glance, the accessibility to the glycosylated residues can be an indicator of non-embedded residues in the loop (i.e., asparagines). At least two glycosylated forms of hNET are known with molecular weights of 80 kD and 54 kD corresponding to a core 46k D hNET protein (Melikian et al., 1994). It is neither clear whether all three asparagines are accessible for glycosylation nor if indeed there is a single conformation of this loop. Mutant hNET (K189H) exhibits a zinc binding site in the EL2 loop (Norregaard et al., 1998), which might suggest direct proximity and accessibility between K189 and nearest histidine H372. However, no information is available on the proximity of H199 in the EL2 to these residues. Similarly, several sodium and chloride ion binding sites are reported in LeuT, dDAT and human SERT (hSERT; Yamashita et al., 2005; Penmatsa et al., 2013; Wang et al., 2015; Coleman et al., 2016). Ligands binding sites include the central binding site (S1) and secondary binding site (S2) that overlaps the extracellular loop (EL4; Figure 1A). Conserved residues in binding sites described by Koldsø et al. (2015) have nearly the same rotamers except for F317. Low-conserved residues in binding sites described by Andersen et al. (2015) control the selectiveness of hNET and side-chain conformations of these residues can affect the quality of the model (shown in Figure 1A). As mentioned earlier, the side-chains of identical aligned residues exhibit nearly identical rotamers. As many as 53 amino acid residues in hNET that have identity with hSERT and not dDAT can be used from the hSERT structure to complement the rotamers and improve accuracy of structure (Haddad et al., 2016). We recommend that the constructed homology model is validated for selectivity by docking of previously known ligands such as neurotransmitters and inhibitors.

**Table 1:** **Templates for homology modeling of human norepinephrine transporter (hNET) and extracellular domains of anaplastic lymphoma kinase (ALK) and tropomyosin receptor kinase B (TrkB)**.

Target (ID)	Length	Template (PDB IDs)	Identity	Coverage	Main domains	Reference
hNET (P23975)	617	LeuT (2A65)	150/541 (28%)	56-578	All	Yamashita et al. (2005)
		dDAT (4M48)	320/548 (58%)	56-601	All	Penmatsa et al. (2013)
		dDAT (4XPA)	322/547 (59%)	56-601	All	Wang et al. (2015)
		SERT-ts2 (5I6Z)	291/548 (53%)	51-595	All	Coleman et al. (2016)
ALK (Q9UM73)	1620	PTPRM (2C9A and 2V5Y)	25/164 (15%)*	264-427	MAM1	Aricescu et al. (2006); Aricescu et al. (2007)
			21/159 (13%)*	481-636	MAM2	
		MEP1B (4GWM)	19/164 (12%)*	264-427	MAM1	Arolas et al. (2012)
			17/159 (11%)*	481-636	MAM2	
		LDLR (2KRI)	15/37 (41%)	437-473	LDLa	Lee et al. (2010)
TrkB (Q16620)	822	TrkA (2IFG)	89/256(35%)	32-281	Ig-like C2 Type2, LRR	Wehrman et al. (2007)

*Many identities were lost due to position-specific multiple alignment.

## ALK

ALK is a receptor tyrosine kinase originally discovered by chromosomal rearrangement associated with anaplastic large cell lymphoma. ALK was reported to be constitutively activated by gene amplification in several neuroblastoma cell lines (Osajima-Hakomori et al., 2005), while neuroblastoma-specific mutations in ALK were suitable targets for development of several inhibitor therapeutics (Barone et al., 2013). The three classes of ALK mutations include a constitutively active gain-of-function receptor (ligand-independent), kinase dead mutants, and ligand-dependent mutants (Chand et al., 2013). Fusion proteins involving the intracellular kinase domain of ALK cause several types of cancer and has been targeted by kinase inhibitors. In addition, several mutations in ALK kinase domain result in drug resistance (Roskoski, 2013). The advantages of homology modeling of the extracellular domains include accessibility on cell surface and perhaps less cross interaction than kinase inhibitors. *ALK* gene codes for a protein of approximately 1620 residues (Table 1). The crystal structure of the kinase domain shows the interactions of selective drugs with mutant ALK (Sakamoto et al., 2011; Epstein et al., 2012; Huang et al., 2014). All known crystal models of ALK cover the amino acid range 1058–1411 and unfortunately the extracellular structure has not been well studied. The region spanning the extracellular part of the receptor ~266–636 comprises two meprin/A5/mu domains also known as the MAM domains (Figure 1). MAM1 spans 264–427 and MAM2 spans 478–636. Approximately 16 *N*-linked glycosylated sites are recorded sporadically in the extracellular region. Crystal structures of the MAM domain of receptor protein tyrosine phosphatase MU, also known as PTPRM (Aricescu et al., 2006, 2007), and Meprin A beta, also known as MEP1B (Arolas et al., 2012) can serve as multiple templates for the extracellular domain of ALK. Although many sequence identities are lost due to positional alignment (Table 1), the distinct MAM domains of PTPRM and MEP1B are highly similar with ~1.35 Å root-mean- square deviation (RMSD) of Cα atoms and ~1.43 Å RMSD for all atoms. Two loop regions in MAM1 domain require loop modeling (Figure 1B). The former loop region contains conserved tryptophan (W288) very well stacked in the center of the domain. The equivalent tryptophan (W501) in MAM2 was misaligned in the position-specific alignment and must be corrected for proper modeling (Figure 1B). Similar to hNET, glycosylated residues of the MAM domains can be used to check the orientation of side chains as they should be facing solution. A low-density lipoprotein receptor class A (LDLa) domain spans the region 437–473 between the two MAM domains. The LDLR structure (Lee et al., 2010) is the most related homolog (Table 1). At least three conserved aspartic acid residues and one glutamic acid coordinate a calcium ion in the LDLa domain (Figure 1B).

## TrkB

The neurotrophic Trks, assisted by p75 neurotrophin receptor, play an essential role in biology of neurons by mediating neurotrophin-activated signaling. Neurotrophins include nerve growth factor (NGF), brain-derived neurotrophic factor (BDNF), neurotrophin-3 (NT-3), neurotrophin-4/5 (NT-4/5), neurotrophin-6 and neurotrophin-7 (Ultsch et al., 1999). The expression of type 1 (TrkA) and type 2 (TrkB) receptors is associated with favorable and unfavorable primary neuroblastoma patient outcome, respectively (Thiele and Reynolds, 2005). There are four protein isoforms of TrkA reported in the Uniprot database (ID: P04629). The longest is 796 residues in length. Crystal structures cover two large segments of the extracellular; (Robertson et al., 2001; Wehrman et al., 2007), and intracellular TrkA protein (Wang et al., 2012). On the other hand, out of seven alternative isoforms, the canonical isoform of TrkB is 822 residues in length (Table 1). The crystal structures of TrkB cover the intracellular kinase domain (Bertrand et al., 2012) and Ig-like C2 Type 1 domain of the extracellular region (PDB IDs: 1WWB, 1HCF; Ultsch et al., 1999; Banfield et al., 2001). The rest of extracellular segment of TrkB (residues 32–281) can be reconstructed by homology modeling of TrkA template which has ~35% identity (Table 1). The template allows for modeling of two major extracellular domains of TrkB; namely, the leucine rich repeat (LRR) spanning region 92–137, and the Ig-like C2 Type 2 domain spanning the residues 197–281 (Figure 1C). At least two alignment shifts in the LRR and Ig-like C2 type 2 domains require loop modeling. By superposing the three known structures of Ig-like C2 type 1 domains of TrkA, TrkB and TrkC (PDB IDs: 1WWA, 1WWB, and 1WWC, respectively), a divergent loop spanning TrkB 334–341 region is highlighted (Figure 1C). Along with this loop, several divergent residues might also play role in the selectivity of these Trks to different neurotrophins. However, further work is required to identify the exact binding sites of different neurotrophins (Ultsch et al., 1999). In fact, the network of Trk-neurotrophin is more complex if we assume that either Ig-like C2 Type 2 and LRR domains would be involved in neurotrophins interactions. Intensive work in homology modeling, molecular docking and molecular dynamics is required to shed the light on this network. Similar to the situation in hNET and ALK, inhibitors targeting several Trk receptors at the same time result in targeting of several pathways and lead to several drug side effects. Understanding the selectivity of Trks receptors will play significant role in development of therapeutics.

## Conclusions and Perspectives

Homology modeling is one of the first steps in developing therapeutics for new targets. However, as we showed here the first steps are often crucial and detrimental in constructing new homology structures, not to mention in developing new drugs. Although the rules of “good modeling practice” are not written yet, many lessons can be learned by evaluation of the model and correcting/avoiding errors at early stages of modeling. On the other hand, a bad quality template will not give a good quality homology model. The quality and identity of the template(s) are very important issues. Low-conserved regions in the protein target often play a significant biological role. They require more focus in homology modeling and in a design of new therapeutics.

Several strategies for neuroblastoma therapy have been advancing in parallel in the past few decades. Targeting the cell surface molecules is a strategy that allows for distribution of efforts. The distribution of efforts is defined by effective research management where tasks are distributed among researchers to produce more efficient therapeutics. Indeed, novel cancer therapeutics comprise complexes of several agents that carry out several functions. The selective targeting agent (e.g., peptide or antibody) delivers the cargo (i.e., toxic agent) to the cancer cell, which also requires a carrier (soluble/releasing agent) or a membrane penetrating agent. In this perspective article, the modeler has a more focused objective, which is to develop a “selective” targeting molecule. We hope that these strategies will produce more adaptable, efficient and personalized therapeutics in the future.

## Author Contributions

All authors contributed to the design of work. YH wrote the manuscript. ZH reviewed the manuscript, and VA was principle investigator and contributor to scheme and organization of work.

## Funding

We gratefully acknowledge the Czech Agency for Healthcare Research, AZV (15-28334A) and Ministry of Education, Youth and Sports of the Czech Republic under the project CEITEC 2020 (LQ1601) for financial support of this work. The computational resources were provided by the CESNET LM2015042 and the CERIT Scientific Cloud LM2015085, provided under the programme “Projects of Large Research, Development and Innovations Infrastructures”.

## Conflict of Interest Statement

The authors declare that the research was conducted in the absence of any commercial or financial relationships that could be construed as a potential conflict of interest.
